# Subchondral pressures and perfusion during weight bearing

**DOI:** 10.1186/s13018-020-01754-y

**Published:** 2020-06-29

**Authors:** Michael Beverly, Barbara E. Marks, David W Murray

**Affiliations:** Nuffield Department of Orthopaedics, Rheumatology & Musculoskeletal Sciences, University of Oxford, Botnar Research Centre, Nuffield Orthopaedic Centre, Headington, Oxford, OX3 7LD UK

**Keywords:** Intraosseous pressure, Subchondral perfusion, Hydraulic, Bone fat, Marrow, Valve

## Abstract

**Background:**

Joints withstand huge forces, but little is known about subchondral pressures and perfusion during loading. We developed an in vitro calf foot model to explore intraosseous pressure (IOP) and subchondral perfusion during weight bearing.

**Methods:**

Freshly culled calf forefeet were perfused with serum. IOP was measured at three sites in the foot using intraosseous needles, pressure transducers, and digital recorders. IOP was measured during perfusion, with and without a tourniquet and with differing weights, including static loading and dynamic loading to resemble walking.

**Results:**

IOP varied with perfusion pressure. Static loading increased subchondral IOP whether the bone was non-perfused, perfused, or perfused with a proximal venous tourniquet (*p* < 0.0001). Under all perfusion states, IOP was proportional to the load (*R*^2^ = 0.984). Subchondral IOP often exceeded perfusion pressure. On removal of a load, IOP fell to below the pre-load value. Repetitive loading led to a falling IOP whether the foot was perfused or not.

**Conclusion:**

Superimposed on a variable background IOP, increased perfusion and physiological loading caused a significant increase in subchondral IOP. Force was thereby transmitted through subchondral bone partly by hydraulic pressure. A falling IOP with repeat loading suggests that there is an intraosseous one-way valve. This offers a new understanding of subchondral perfusion physiology.

## Introduction

Intraosseous pressure (IOP) has been studied by authors interested in bone circulation, bone diseases, and bone pain for more than 70 years [1–4]. Measurement techniques have varied, and there has been difficulty in establishing a reliable value for IOP [5, 6]. It is generally assumed that IOP is due to venous back pressure or an intrinsic tissue pressure [7–9]. There has been limited progress in understanding IOP and subchondral bone perfusion physiology since Azuma reported IOP fluctuation in a rabbit model in 1964 [10]. IOP has often been found to be raised in bone diseases such as osteonecrosis and after steroid use. A raised IOP has been associated with pain in osteoarthritic joints, chondromalacia patellae, and with cartilage degeneration [11–13]. Ficat developed a technique for the “functional exploration” of bone in patients with early osteonecrosis [14]. In man, clinical measurement of IOP has offered variable results [15], while measuring IOP pulsation and a respiratory wave have been reported [16]. Studies by Freeman and Swanson considered the possibility that bone was hydraulically strengthened [17, 18] but hydraulic pressure transmission was discounted. More recently, Simkin suggested that trabeculae and fat act together to cushion the effects of weight bearing in the subchondral region [19]. Although the skeleton is clearly designed for weight bearing, the possibility that IOP changes during weight bearing has not been explored. Denham and others calculated that forces of several times body weight are transferred across joints during activity [20]. We noticed that pressure or weight bearing on a limb altered the IOP [21]. We developed an in vitro model to explore IOP and subchondral pressures [22]. We used the model to study perfusion physiology with combinations of static and dynamic loading.

## Methods

### Model preparation

**Fig. 1:**
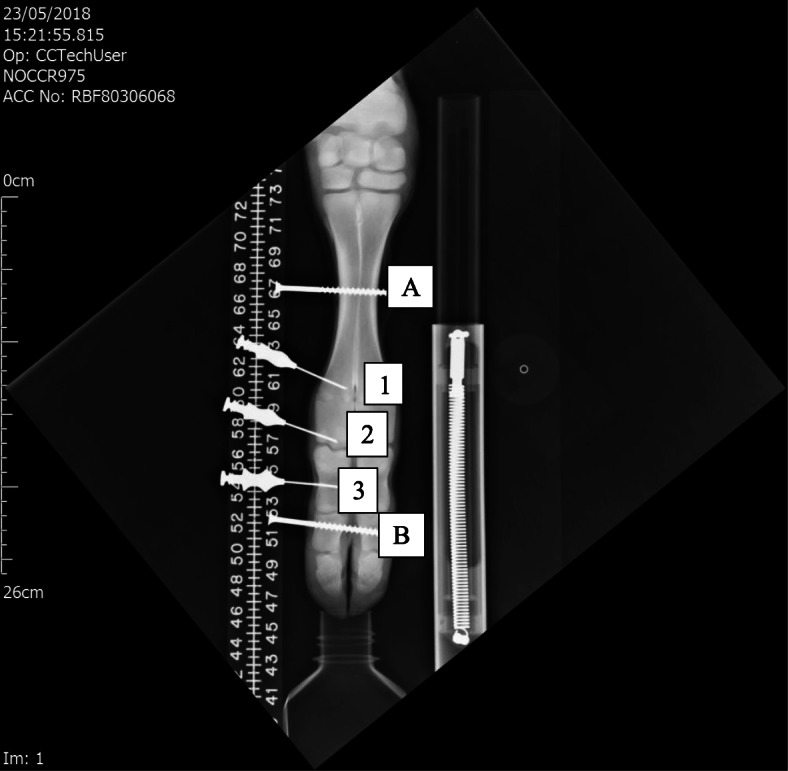
Experimental set up showing the Ex-fix screws labeled A in the metacarpal diaphysis and B in the middle phalanx. IOP needles are seen in the metacarpal metaphysis [23], subchondral epiphysis [1], and middle of the proximal phalanx [10]. The loading device is seen on the right with its calibrated internal spring

Forefeet from freshly culled Friesian male calves weighing 50–80 kg were used. The radial artery was catheterized with a 16-gauge 12" Teflon catheter (E-Z Cath, Desert Pharmaceutical, UT, USA), and the artery was rinsed with 120 ml of warmed Krebs-Henseleit (K-H) solution (Sigma Pharmaceuticals, Watford, UK). Feet were placed in a water bath within 1 h post-mortem. A circulating pump (Fluval FLU.U1 model C-48020, Rolf C. Hagen Ltd., Castleford, UK) and a thermostat-controlled heater (Aqua One 100-Watt IPX8, Aqua Pacific UK Ltd., Southampton, UK) with a room thermometer-maintained temperature within a degree of 37° C were used. A doubled string tourniquet was applied at the carpus above the metacarpal. The arterial catheter tip lay distal to the tourniquet. Calf serum was prepared by centrifuging blood at 10,000 rpm for 10 min at 4°C. The serum was re-warmed through an IV line in the water bath before entering the foot. Serum was perfused from a height using a Mariotte flask syphon to give a steady pressure of 50, 100, or 150 cm of water. These heights equate to pressures of 37, 74, and 110 mmHg. Intraosseous needles (Rosenthal Bone Biopsy Needle, Luer lock fitting, 1.6 mm × 35 mm length, Dixons Surgical Instruments Ltd., Essex, UK) were inserted by hand percutaneously into the distal metacarpal subchondral bone, the metacarpal epiphysis, and in the mid proximal phalanx (Fig. 1). The needles were connected to catheter tip pressure transducers (Model TT Luer, Gaeltec Devices Ltd., Isle of Skye, UK). The transducers were connected to a Gaeltec S7d amplifier and an ADC-20 Picolog Data Logger (Pico Technology, Saint Neots, UK).

Data was recorded using a Picolog Recorder program on a ProDesk Hewlett-Packard PC. The transducers were calibrated against a 100-mmHg sphygmomanometer and the system was zeroed before each run. The millivolt recorded values were converted to mmHg.

In all records, the first of the four channels was used for recording the perfusion pressure at the entry to the arterial catheter. IOP recordings were made from the other three intraosseous needles simultaneously, one per second for up to an hour.

### Loading method–external fixator

For external fixator (Ex-fix) loading, screws were placed across the middle of the metacarpal and the middle of the middle phalanx (Fig. 1). A “scissor” type external fixator was attached to the screws on both sides of the foot. Compression was applied to the fixator arm ends through a fisherman’s scale with a 0–20 kg range. The screw to hinge (10 cm) and hinge to lever arm (35 cm) distance ratio gave a 3.5 multiplication factor for the force across the joint. With Ex-fix loading, the force across the joint was purely skeletal without movement or compression of the soft tissues.

### Loading method–physiological standing

“Standing” load on the tip of the hoof caused the foot to adopt a posture of slight hyperextension as seen in the natural posture of the calf. By X-ray and direct measurement, the distance from the center of rotation at the joint to the center of the posterior restraining or tension elements was 15 mm, and the distance from the center of rotation at the joint to the weight-bearing line in front of the joint calculated on a stress X-ray was 30 mm. A factor of twice the applied longitudinal load was therefore transmitted across the joint surfaces. The same proportional formula applied whatever the size of the foot.

### Experimental plan

Because our earlier work had shown a relationship between IOP and perfusion or obstruction of proximal arterial or venous drainage supply, for this work, we designed three different perfusion regimes. Perfusion was either “no perfusion,” “perfusion at 150 cm pressure,” or “perfusion at 150 cm with a tourniquet” placed proximal to the arterial catheter tip. The polythene arterial catheter was not crushed or closed by the tourniquet, but venous drainage was prevented.

Loading was carried out with either the Ex-fix device or with a physiological standing type of load.

“Static” loads were applied for 10 s separated by 10 s rest intervals.

“Dynamic” loading was applied at 1/s for 10 “steps” with a 10 s rest interval before the next “walk.”

The weights or forces used were 5, 10, 15, and 20 kg. These were multiplied × 3.5 for Ex-fix loading and × 2 for the standing loading.

### Statistics

Results were expressed as means, standard deviations, and ranges. Student’s *t* test was used to determine if there were significant differences. When each subject was used as its own control, paired tests were used. Otherwise unpaired tests were used. The Pearson test was used to assess correlations, and *p* < 0.05 was considered to be statistically significant.

## Results

### IOP with loading non-perfused, perfused, or perfused and with a tourniquet

**Fig. 2:**
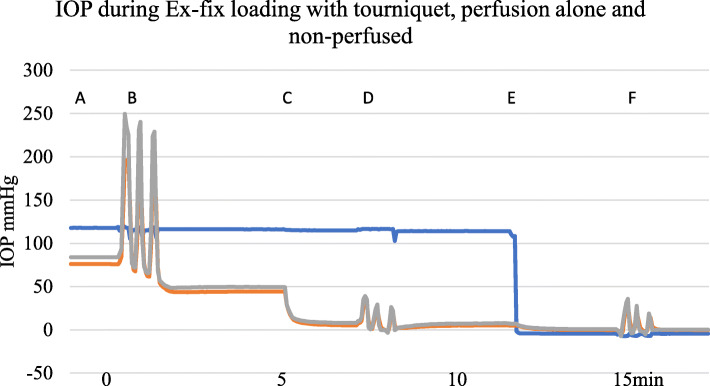
Example showing A—IOP during perfusion with a proximal tourniquet. B—with Ex-fix loading of 10 kg for 10 s with 10 s intervals × 3 during perfusion with a tourniquet in place, C—removal of the tourniquet at 5 min, D—loading during perfusion alone at 7 min, E—cessation of perfusion at 12 min, and E—loading when non-perfused at 15 min. Blue—serum perfusion pressure, red—metacarpal diaphysis IOP, green—metacarpal epiphyseal IOP

Initial IOP measurements varied between and within subjects. There were 15 subjects with 40 different IOP recording sites. After 60 s of perfusion at 150 cm pressure, there was a mean IOP of 30.0 mmHg (SD 14.4, range 7.6 mmHg to 52.7 mmHg). Changes in IOP with perfusion took place over the course of approximately a minute whereas physical loading either by Ex-fix or by standing caused an immediate effect on IOP. IOP changed with load whether or not the foot was being perfused. Figure 2 shows a typical trace during Ex-fix loading with perfusion and a tourniquet, perfusion alone, and loading without perfusion.

In a group of nine separate feet, the effect on IOP of Ex-fix loading to 10 kg across the joint was a significant rise in IOP whatever the perfusion conditions (Table 1).

**Table 1:** IOP without perfusion and no load or 10 kg load, perfused at 150 cm no load and 10 kg load and perfused with a proximal tourniquet

	IOP no load	IOP with 10 kg load	
Non perfused	− 0.1 (0.8)	23.8 (4.5)	*p* < 0.0001
Perfused at 150 cm	10.7 (1.4)	62.9 (9.6)	*p* < 0.0001
Perfused plus tourniquet	50.0 (4.4)	151 (15.2)	*p* < 0.0001

*N* nine feet (SEM)

### IOP proportional to the load

**Fig. 3:**
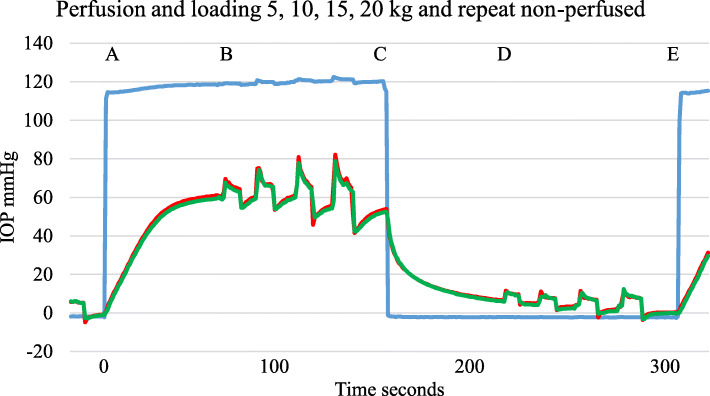
Example trace. A—perfusion for 60 s, then B—standing type static load for 10 s at 5, 10, 15, and 20 kg with 10 s between, C—perfusion off for 60 s then D—repeat load pattern non-perfused. E—perfusion restarted. Blue—perfusion pressure, red—metacarpal diaphysis IOP, green—metacarpal epiphysis IOP

Loads of 5, 10, 15, and 20 kg caused a proportionate increase in IOP from the background non-perfused or perfused IOP. Figure 3 shows a typical graph with Ex-fix loading at 5, 10, 15, and 20 kg for 10 s during perfusion and without perfusion.

**Fig. 4:**
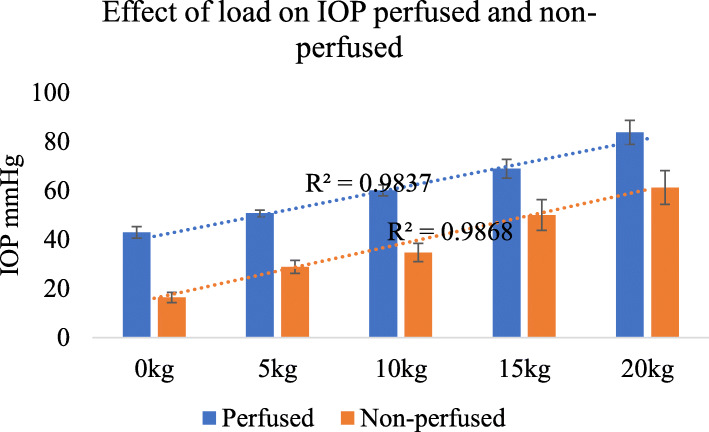
IOP was proportional to the applied load in both the non-perfused state and the perfused state *n* = 22 different sites, error bars SEM

Although there was a wide variation in the initial IOP, at any one site, there was a close correlation between the load applied and the rise in IOP in both perfused and non-perfused feet (Fig. 4).

### IOP and load with respect to the perfusion pressure

**Fig. 5:**
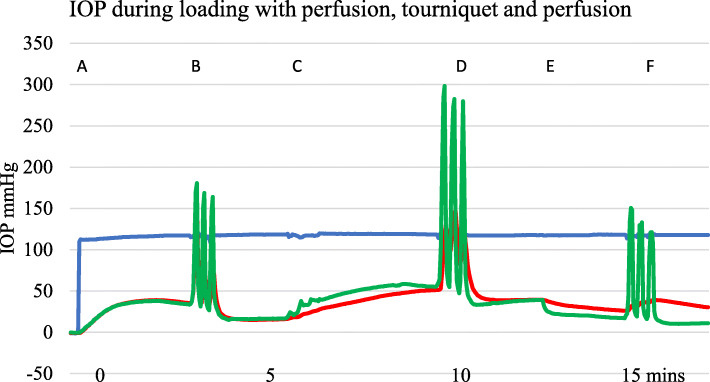
Example of IOP with standing type loads. A—perfusion starts at 150 cm pressure. B—loading during perfusion with a static load of 10 kg applied for 10 s with 10 s interval × 3. C—tourniquet applied, D—further loading, E—tourniquet removed, and F—loading during perfusion again. Blue—serum perfusion pressure, red—metacarpal diaphysis IOP, green—metacarpal epiphysis IOP

**Fig. 6:**
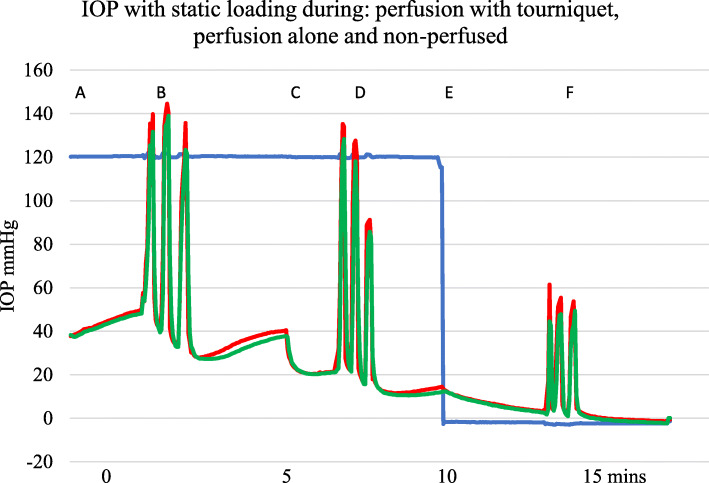
Example showing effect of three sets of 10 kg standing loads for 10 s with 10 s intervals. A—IOP perfused with tourniquet applied, B—loading cycles, C—tourniquet removed at 5 min, D—loading during perfusion, E—perfusion stopped, and F—loading while non-perfused. Blue—serum perfusion, red—metacarpal diaphysis IOP, green—metacarpal epiphysis IOP. There is a progressive fall in IOP after removal of the load in all three perfusion states

An example of a trace with perfusion and Ex-fix loading of 10 kg for 10 s with and without a proximal venous tourniquet is shown in Fig. 5. IOP can exceed the perfusion pressure as seen in Figs. 2, 5, and 6.

### Effect of removal of load

Whatever the perfusion state, a reduced IOP is seen after removal of a standing load (Figs. 5 and 6). With repeated loading, there appears to be a more obvious pressure loss implying a “valve” or “pump out” effect lowering IOP.

### Dynamic loading

#### Repetitive loading to mimic walking

**Fig. 7:**
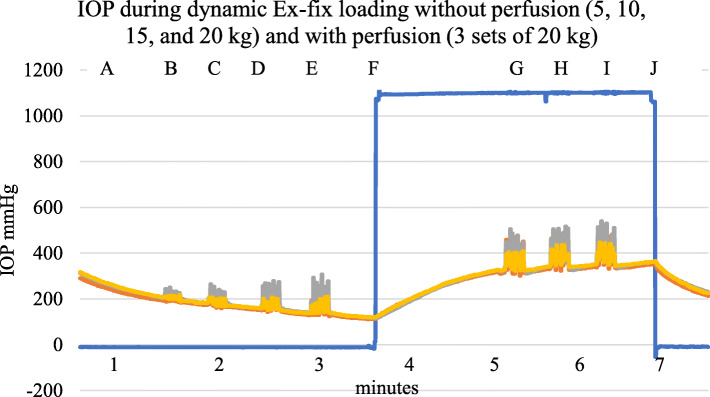
Example of a typical dynamic Ex-fix load trace. A—IOP while non-perfused, B to E—5, 10, 15, and 20 kg load of 10 steps in 10 s, F—perfusion commenced, G, H, I—20 kg for 10 steps in 10 s, J—perfusion stopped. Blue—perfusion pressure 0–150 cm, red—metacarpal diaphysis IOP, green—metacarpal subchondral IOP, purple—proximal phalanx diaphysis IOP

Loading by external fixator to simulate walking at one second intervals for 10 s produced a fluctuating IOP with or without perfusion, proportional to the applied load as shown in Fig. 7.

#### Repeated dynamic loads under different perfusion conditions

Repetitive loading with an Ex-fix was applied, with and without perfusion. The IOP fluctuated whether in the perfused state or not (Fig. 7). In the standing or more physiological type of load, there was increased emptying with activity (Fig. 6).

**Fig. 8:**
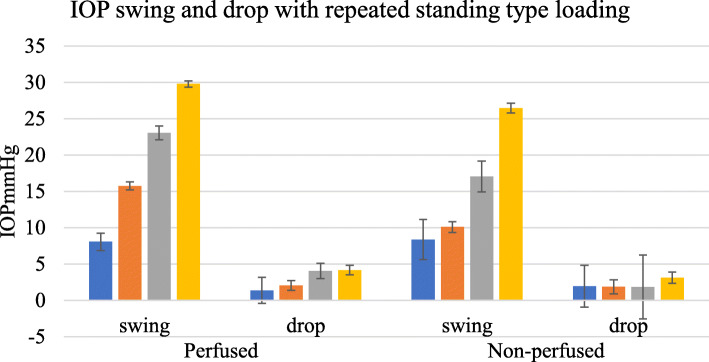
IOP fluctuation with loading. Blue—5 kg, red—10 kg, green—15 kg, and purple—20 kg loads, 10 steps in 10 s and associated drop in IOP post-exercise for perfused and non-perfused feet, *n* = 24 sites in 11 feet, error bars SEM

The “swing” or fluctuation with physiological or standing type load was compared in the perfused and non-perfused state in 24 sites among 11 feet. The swing was defined as the peak to trough excursion with the application and removal of load. With 10 standing type steps, there was a swing proportional to the load. The “drop” was defined as the difference between the starting IOP and the IOP after 10 steps. This is shown for both the perfused and non-perfused states (Fig. 8).

## Discussion

### IOP measurement

The wide variation found in single IOP measurements under apparently similar circumstances has been reported by other authors [15]. Previously, it was assumed that normal bone has an IOP that is constant. But it is only in bone that pressure has been measured by inserting a needle into a solid organ. Measurements of pressure elsewhere in the body rely on vessel catheterization or pressure measurement within spaces such as in the brain or bladder. We were unable to find previous work offering an explanation for IOP variation under similar experimental conditions [22, 24, 25]. We consider that our findings together with observations made by previous authors point to there being an alternative explanation [7, 14, 16, 23, 24, 26]. The variation in IOP is probably not due to a significant physiological difference between subjects but to the random nature of the vessels contacted at the needle tip, even with standardized needle insertion. Where a small artery is encountered a relatively high IOP and pulse volume might be recorded. Where capillaries or veins, fat and trabeculae are encountered then a lower IOP with minimal pulse volume is found. We consider that the widespread distribution of values found despite a similar technique supports this interpretation. It also means that a single measurement of IOP is of little clinical value. Fortunately, that does not detract from using IOP to explore subchondral physiology with perfusion and weight bearing as in this work.

### Rate of change in IOP

Our results demonstrate that while IOP changes slowly over the course of about a minute with perfusion changes, IOP changes immediately with application or removal of a load, whatever the perfusion state. The bone IOP appears to respond instantly to compression whereas background IOP reacts more slowly to perfusion filling or emptying the whole foot.

### IOP proportional to load

In spite of a wide range of initial IOP values, there is a proportional increase in IOP with loading, whatever the perfusion state. This appears to suggest that there is a proportional elastic intraosseous hydraulic pressure or hydrodynamic response to loading of bone over the physiological range tested here. We were unable to find any previous reference confirming hydraulic pressure transmission in the bone [18].

### Perfusion state and IOP

The response of IOP to load is least where the bone is non-perfused and greatest when the bone is perfused and with a proximal tourniquet. This appears to be due to simple hydrodynamics. When non-perfused the subchondral bone IOP responds less to load but when full or charged, a greater IOP is generated by the same load. In the first instance, the cancellous bone is relatively void or empty but with perfusion with a proximal tourniquet in place the cancellous bone is charged or full and pressurized, and the same load generates a proportionately higher IOP.

### IOP above perfusion pressure

As seen in several traces, IOP with loading may exceed the circulating or perfusion pressure. Others have calculated that, in use, surface forces of up to 20,000 mmHg might be experienced [20, 23, 26]. Our pressure transducers were restricted to 300 mmHg. If IOP reaches 20,000 mmHg in vivo, there are likely to be anatomical and physiological modifications to cope [27].

### Hydraulic force transfer

The close relationship between load and a proportional increase in IOP appears to confirm that this is a simple hydrodynamic or elastic model. We suggest that force is transferred from the soft cartilaginous joint surface through the slightly flexible subchondral bone plate and into the underlying perfused soft fatty cancellous bone tissue. Bone lipid is almost liquid at body temperature (personal observation) as seen during bone surgery or with fat/fluid levels on lateral X-rays of fractures. Bone fat is usually contained within delicate adipocytes [28]. It does not flow but could behave as a fluid in terms of transferring hydraulic force. The raised subchondral IOP transfers force by hydraulic pressure on to the trabeculae. The trabeculae in turn coalesce towards the cortex and transfer the force to the more rigid cortical shaft and down the bone. The process is reversed at the next joint. Although previous authors considered the possibility of hydraulic forces within bone, they were using non-physiological methods with grease saturated dry bones or cancellous bone specimens in jigs [17, 18].

### Type of loading

Two different methods of loading were used. Purely skeletal loading through an Ex-fix caused a simple rise and fall in subchondral IOP as might be expected. This applied whether the load was static for 10 s or a more dynamic repeated load at 1/s. The more physiological standing or hoof tip type of load caused an apparent fall in IOP to below the starting point after the hoof tip load was removed. The fall was present whatever the perfusion state. If the system is hydraulic and fully elastic, removal of the load would be expected to result in a fall in pressure, but only to around the starting point. Furthermore, any loss in hydraulic elasticity should result in a lesser fall in IOP, that is, to above the start point.

With standing loads, there is a posterior “calf muscle” type of venous pump in the calf foot similar to that in man (Gardner and Fox 1989). There is also a hoof venous sinus which is compressed and empties blood during the stance phase in the hoof [29]. None of these apply with the use of the pure skeletal compression produced by the Ex-fix. Blood is returned up the valved venous system towards the heart. As a result, the extra osseous venous pressure falls. After load is removed, the reduced pressure seen in the IOP needle probably reflects the reduced extra osseous soft tissue pressure. It is also possible that an intraosseous or cortical level “one-way valve” exists designed to facilitate subchondral osseous blood flow by active emptying of bone blood with exercise. “Topping-up” of the system takes place normally between steps when perfusion is present from the normal low-pressure circulation at rest. With exercise, much higher pressures operate to transfer load to the cortex by hydraulic pressure.

## Limitations

There are several possible limitations in this work. The calves were all male Friesian animals of similar but not identical age and weight. They were probably more similar than any group of patients might be. The bone was of a juvenile pattern with cartilaginous epiphyses present. Flow across the epiphyses is probably minimal, but IOP changes were seen in all areas tested. The calf has a fused third and fourth metacarpal which becomes bifid at the distal metacarpal and the phalanges and hoof as seen in Fig. 1. The specimens resemble the adult forearm and wrist in size, weight, and bone strength. The fore feet were catheterized through the equivalent of the radial artery and rinsed out. The depth of insertion of the catheter varied but was to about 6" or well into the proximal third of the metacarpal. Flushing was carried out by hand and irrigation pressures may have varied. The emerging perfusate was almost but not completely clear of blood. Needle insertion was by hand and sites could not be identical. Needle placement was within a few millimeters of the subchondral surface or the central diaphysis. The loading methods used were relatively crude. The application of Ex-fix screws through the foot might be expected to alter pressures within the bone. A bone segment was "skipped" for the distal Ex-fix attachment, and IOP was then tested in the unscathed metacarpal epiphysis and proximal phalanx. It could be argued that any penetration of the skin and bone with drills, screws, or needles was damaging to the integrity of the specimen. However, when no bone screw was used, such as in the more physiological hoof tip loading, the IOP results and responses were similar to those with an Ex-fix. Beyond 300 mmHg pressure transducer overload occurred. The color of the serum varied between batches from straw color to a medium hemoglobin pink. Despite practice with insertion, connection, and setting up of the pressure transducers, the experiment took time and could not be guaranteed to work on every occasion. Of all attempted recordings, some 80% were successful. Leakage, obstruction, bubbles and unknown transducer, recorder, and data collection failures account for the others. All useable records were included. No outliers were excluded.

## Conclusion

Our 3Rs compliant in vitro model shows that irrespective of the initial IOP, there is a close correlation between subchondral IOP, perfusion pressure, and load. High pressures are generated by load bearing. Load is transferred from the subchondral region partly by hydraulic pressure to the trabeculae. This study opens a new field of physiology and may offer a window to understanding subchondral circulation and joint function in bone disease such as osteoarthritis.

## Data Availability

The authors will give access to all data if requested

## Abbreviations

IOP: Intraosseous pressure

## References

[1] Arnoldi CC, Linderholm H, Mussbichler H (1972). Venous engorgement and intraosseous hypertension in osteoarthritis of the hip. J Bone Joint Surg Br.

[2] Barclay AE (1948). Micro-Arteriography. American Journal of Roentgenology.

[3] Crock HV (1980). An Atlas of the arterial supply of the head and neck of the femur in man. Clin Orthop Relat Res.

[4] Trueta J, Harrison MHM (1953). The Normal Vascular Anatomy of the Femoral Head in Adult Man. Journal of Bone and Joint Surgery-British Volume.

[5] Hungerford DS, Lennox DW (1985). The importance of increased intraosseous pressure in the development of osteonecrosis of the femoral head: implications for treatment. Orthop Clin North Am.

[6] Wilkes CH, Visscher MB Some physiological aspects of bone-marrow pressure. J Bone Joint Surg Br.1975 A 57 1. 49-57. 1123371

[7] Green NE, Griffin PP (1982). Intra-osseous venous pressure in Legg-Perthes disease. J Bone Joint Surg Am.

[8] Lemperg RK, Arnoldi CC (1978). The significance of intraosseous pressure in normal and diseased states with special reference to the intraosseous engorgement-pain syndrome. Clin orthop.

[9] Owen R, Goodfellow J, Bullough PG. Scientific foundations of orthopaedics and traumatology. London: William Heinemann; 1980. xii, 531 p. p.

[10] Azuma H (1964). Intraosseous pressure as a measure of hemodynamic changes in bone marrow. Angiology..

[11] Liu SL, Ho TC (1991). The role of venous hypertension in the pathogenesis of Legg-Perthes disease. A clinical and experimental study. J Bone Joint Surg Am.

[12] Termansen NB, Teglbjaerg PS, Sorensen KH (1981). Primary osteoarthritis of the hip. Interrelationship between intraosseous pressure, X-ray changes, clinical severity and bone density. Acta Orthop Scand.

[13] Uchio Y, Ochi M, Adachi N, Nishikori T, Kawasaki K. Intraosseous hypertension and venous congestion in osteonecrosis of the knee. Clin Orthop Relat Res, 384. 2001:217–23. 10.1097/00003086-200103000-00025 11249168

[14] Ficat RP (1985). Idiopathic bone necrosis of the femoral head. Early diagnosis and treatment. Journal of Bone and Joint Surgery-British Volume.

[15] Salzman JG, Loken NM, Wewerka SS, Burnett AM, Zagar AE, Griffith KR (2017). Intraosseous Pressure Monitoring in Healthy Volunteers. Prehospital Emergency Care.

[16] Beverly M, Urban J, Murray D (2016). Factors affecting physiology of intraosseous pressure measurement. Osteoarthritis Cartilage.

[17] Hwa HJ (2004). Could the intraosseous fluid in cancellous bone bear external load significantly within the elastic range?. Proc Inst Mech Eng H.

[18] Swanson SA, Freeman MA (1966). Is bone hydraulically strengthened?. Med Biol Eng.

[19] Simkin PA. Marrow fat may distribute the energy of impact loading throughout subchondral bone. Rheumatology (Oxford). 2017. 10.1093/rheumatology/kex274 PMC5850356 28977578

[20] Denham RA (1959). Hip mechanics. Journal of Bone and Joint Surgery-British Volume..

[21] Beverly M, Pflug J, Mathie R. Bone - a flexible perfused sponge. Journal of Bone and Joint Surgery-British Volume 1987. p. 494-.

[22] Beverly M, Murray D (2019). An in vitro model to explore subchondral perfusion and intraosseous pressure. Journal of Experimental Orthopaedics.

[23] Afoke NY, Byers PD, Hutton WC (1987). Contact pressures in the human hip joint. Journal of Bone and Joint Surgery-British Volume.

[24] Beverly M, Mellon S, Kennedy JA, Murray DW (2018). Intraosseous pressure during loading and with vascular occlusion in an animal model. Bone & Joint Research.

[25] Beverly M, Murray D (2018). Factors affecting intraosseous pressure measurement. J Orthop Surg Res.

[26] Fukubayashi T, Kurosawa H. The contact area and pressure distribution pattern of the knee: a study of normal and osteoarthrotic knee joints. Acta orthopaedica Scandinavica, 1980. 51(1-6):871–9. 10.3109/17453678008990887 6894212

[27] Beverly M (2019). The Role of Subchondral Circulation in the Physiology of Load Transmission [DPhil in Musculoskeletal Science].

[28] Bryant J, David T, Gaskell P, King S, Lond G Rheology of bovine bone marrow. Proceedings of the Institution of Mechanical Engineers, Part H: Journal of Engineering in Medicine.1989 203 2. 71-75. 10.1243/PIME_PROC_1989_203_013_01 2487507

[29] Robinson N (1990). Digital blood flow, arteriovenous anastomoses and laminitis. Equine Veterinary Journal.

